# Neuromechanisms of SARS-CoV-2: A Review

**DOI:** 10.3389/fnana.2020.00037

**Published:** 2020-06-16

**Authors:** Marcos F. DosSantos, Sylvie Devalle, Veronica Aran, Daniela Capra, Natália Roberta Roque, Juliana de Mattos Coelho-Aguiar, Tânia Cristina Leite de Sampaio e Spohr, Janice Gonçalves Subilhaga, Cláudia Maria Pereira, Isabella D'Andrea Meira, Paulo Niemeyer Soares Filho, Vivaldo Moura-Neto

**Affiliations:** ^1^Laboratório de Morfogênese Celular, Instituto de Ciências Biomédicas, Universidade Federal do Rio de Janeiro, Rio de Janeiro, Brazil; ^2^Programa de Pós-Graduação em Medicina (Radiologia), Universidade Federal do Rio de Janeiro, Rio de Janeiro, Brazil; ^3^Programa de Pós-Graduação em Neurociência Translacional, Instituto Nacional de Neurociência Translacional (INNT-UFRJ), Rio de Janeiro, Brazil; ^4^Laboratório de Biomedicina do Cérebro, Instituto Estadual do Cérebro Paulo Niemeyer, Secretaria de Estado de Saúde, Rio de Janeiro, Brazil; ^5^Programa de Pós-Graduação em Anatomia Patológica, Hospital Universitário Clementino Fraga Filho, Universidade Federal do Rio de Janeiro, Rio de Janeiro, Brazil; ^6^Setor de Pneumologia, Serviço de Clínica Médica, Hospital Federal dos Servidores do Estado, Rio de Janeiro, Brazil; ^7^Programa de Pós-Graduação em Biomedicina Translacional e Odontologia Clínica e Experimental, Universidade do Grande Rio (Unigranrio), Duque de Caxias, Brazil; ^8^Departamento de Neurologia, Universidade Federal Fluminense, Niterói, Brazil; ^9^Programa de Epilepsia do Instituto Estadual do Cérebro Paulo Niemeyer, Secretaria de Estado de Saúde, Rio de Janeiro, Brazil; ^10^Instituto Estadual do Cérebro Paulo Niemeyer, Secretaria de Estado de Saúde, Rio de Janeiro, Brazil

**Keywords:** SARS-CoV-2, COVID-19, central nervous system, peripheral nervous system, anosmia, dysgeusia

## Abstract

Recent studies have suggested the neuroinvasive potential of severe acute respiratory coronavirus 2 (SARS-CoV-2). Notably, neuroinvasiveness might be involved in the pathophysiology of coronavirus disease 2019 (COVID-19). Some studies have demonstrated that synapse-connected routes may enable coronaviruses to access the central nervous system (CNS). However, evidence related to the presence of SARS-CoV-2 in the CNS, its direct impact on the CNS, and the contribution to symptoms suffered, remain sparse. Here, we review the current literature that indicates that SARS-CoV-2 can invade the nervous system. We also describe the neural circuits that are potentially affected by the virus and their possible role in the progress of COVID-19. In addition, we propose several strategies to understand, diagnose, and treat the neurological symptoms of COVID-19.

## Introduction

A highly pathogenic coronavirus termed severe acute respiratory syndrome coronavirus 2 (SARS-CoV-2) emerged in December 2019 in Wuhan, China. Since then, it has rapidly spread globally. SARS-CoV-2 causes coronavirus disease 2019 (COVID-19), which can result in rapid and intense respiratory symptoms and lung failure (Dong et al., 2020; Park et al., 2020). Genomic analysis revealed that this virus belongs to the same clade, beta coronaviruses, as severe acute respiratory syndrome coronavirus (SARS-CoV) and Middle East respiratory syndrome coronavirus (MERS-CoV) and that it shares a highly homologous sequence with SARS-CoV (Yu et al., 2020). Due to its high infectivity, the number of people with COVID-19 continues to grow worldwide.

Epidemiological studies have shown that patients with COVID-19 can present a number of symptoms, including a broad range of neurological complications such as headache, ischemic stroke, impaired consciousness, and encephalitis/meningitis (Gautier and Ravussin, 2020; Giacomelli et al., 2020; Mao et al., 2020; Poyiadji et al., 2020; Wang D. et al., 2020 (Table 1). Moreover, the presence of olfactory and taste disorders such as anosmia, hyposmia, ageusia, and dysgeusia in many individuals suggests the involvement of cranial nerves (Gautier and Ravussin, 2020; Giacomelli et al., 2020). Possible involvement of the nervous system (NS) structures, particularly the brainstem, by SARS-CoV-2, has also been suggested (Li Y. C. et al., 2020). According to this hypothesis, which is based on older articles (Andries and Pensaert, 1980; Matsuda et al., 2004; Li et al., 2012, 2013), coronaviruses initially invade peripheral-nerve terminals, and then progress toward the central nervous system (CNS), via synapse-connected routes. This would not be surprising, given the capacity of different viruses (e.g., Herpes Simplex and Herpes Zoster) to migrate via peripheral neurons to either sensory ganglia or to the CNS, where they cause extensive damage (usually after dormancy reactivation) (Oaklander, 2001; Watson and Oaklander, 2002; Dworkin et al., 2008; Dasilva and Dossantos, 2012). Interestingly, a study used electron microscopy to explore the dissemination of the hemagglutinating encephalomyelitis virus (HEV), also a single-stranded RNA beta coronavirus, in the primary motor cortex of infected rats (Li et al., 2013). The results revealed that membranous coating-mediated endo-/exocytosis might be used for the transsynaptic exchanges of coronaviruses. The same study indicated that the transsynaptic pathway could be adapted for larger granular material, including viruses (Li et al., 2013).

**Table 1:** Percentages of patients with COVID-19 that suffered from neurological symptoms, considering the studies included in the current review.

**Main neurological symptoms**	**Percentage (Reference)**
Acute cerebrovascular disease	2.8% (Mao et al., 2020)
Acute hemorrhagic necrotizing encephalopathy	Case report (Poyiadji et al., 2020)
Ataxia	0.5% (Mao et al., 2020)
Ageusia	1.7% (Giacomelli et al., 2020)
Ageusia and anosmia	8.5% (Giacomelli et al., 2020)
Ageusia and hyposmia	3.4% (Giacomelli et al., 2020)
Dizziness	9.4% (Wang D. et al., 2020); 16.8 (Mao et al., 2020)
Dysgeusia	8.5% (Giacomelli et al., 2020)
Dysgeusia and anosmia	3.4% (Giacomelli et al., 2020)
Dysgeusia and hyposmia	3.4% (Giacomelli et al., 2020)
Headache	3.4% (Giacomelli et al., 2020); 6.5% (Wang D. et al., 2020); 13.1% (Mao et al., 2020)
Hyposmia	5.1% (4); 5.1% (Mao et al., 2020)
Impaired consciousness	7.5% (Mao et al., 2020)
Ischemic stroke	2.8% (Mao et al., 2020)
Meningitis/Encephalitis	Case report (Moriguchi et al., 2020)
Nerve pain	2.3%
Seizure	0.5% (Mao et al., 2020)
Smell impairment	5.1% (Mao et al., 2020)
Taste impairment	5.6% (Mao et al., 2020)
Vision impairment	1.4% (Mao et al., 2020)
Vomiting	3.6% (Wang D. et al., 2020)

Strikingly, coronaviruses have also been reported to cause enteric diseases in both animals and humans (2003; Zhou et al., 2017). Therefore, the involvement of the enteric nervous system (ENS) in the pathophysiology of COVID-19 must also be considered.

This critical review aims to comprehensively evaluate the current literature and examine the available evidence regarding the impacts of COVID-19 on the NS. The possible neural circuits involved in this process and the putative pathophysiological mechanisms will be analyzed. Finally, different approaches to study these phenomena will be proposed.

## Interpretation of the Neuroinvasion Related to SARS-CoV-2 and the Main Neurological Symptoms Associated With COVID-19

In a study exploring the main signs and symptoms found in 138 hospitalized patients diagnosed with COVID-19-related pneumonia, all patients reported persistent olfactory and taste disorders (Wang D. et al., 2020). Remarkably, taste alterations were more frequent before hospitalization (91%), whereas after hospitalization, taste and olfactory alterations had equal frequency. The epidemiological heterogeneity related to the type and time-course of these symptoms suggests that COVID-19 might differentially affect individuals according to factors such as gender and age (Giacomelli et al., 2020). In addition, due to its early onset, these taste and olfactory changes provide a useful and straightforward screening opportunity to identify people with COVID-19 and limit viral spread (Gautier and Ravussin, 2020; Giacomelli et al., 2020). Neurological symptoms were also analyzed in a cohort of 214 hospitalized patients diagnosed with COVID-19 (Mao et al., 2020), which found an incidence of anosmia in 5.1% and ageusia in 5.6%. Anosmia has been previously associated with SARS-CoV and other coronaviruses (Hwang, 2006; Vaira et al., 2020). Moreover, it has been proposed that SARS-CoV-2 enters through the olfactory nerve and reaches the brain, in the same manner as SARS-CoV.

It is likely that the mechanisms underlying the taste and olfactory changes in the course of COVID-19 are related to the role played by the angiotensin-converting enzyme-2 (ACE2) receptor (Lu et al., 2020). It has been demonstrated that this receptor is used by SARS-CoV-2 to bind and penetrate human cells (Lu et al., 2020). The wide expression of the ACE2 receptor in the epithelial cells of the oral and nasal mucosa (Xu et al., 2020) suggests that the virus-related damage caused by SARS-CoV-2 in these sites is linked to the effects of the virus on the function of the ACE2 receptor. However, the specific expression of ACE2 through the olfactory pathway in humans and rodents is still debated and has been explored in several studies (Chen R. et al., 2020; Li Y. C. et al., 2020; Natoli et al., 2020; Ueha et al., 2020; Wu Y. et al., 2020). Moreover, it is important to consider the roles of Furin and the transmembrane protease serine 2 (TMPRSS2) in this process (Ueha et al., 2020). Overall, ACE2 is responsible for the entry of SARS-CoV-2 into cells. This process depends on the binding of the viral spike, glycoprotein, to this cellular receptor. The process also involves cleavage of the viral spike protein by Furin, as well as the occurrence of spike-protein priming by host-cell serine proteases such as the transmembrane protease serine 2 (TMPRSS2), which is localized in the cell membrane and facilitates viral uptake (Li et al., 2003; Coutard et al., 2020; Hoffmann et al., 2020; Lukassen et al., 2020; Matsuyama et al., 2020; Walls et al., 2020; Wu F. et al., 2020). Although there is a lack of evidence regarding the distributions of ACE2, TMPRSS2, and Furin in the different cellular components of the olfactory pathway, the close relationship between smell and the central nervous system (CNS), together with the occurrence of neurological symptoms in patients with COVID-19, alludes to the involvement of the CNS. The possible involvement of the olfactory pathway in the route of SARS-CoV-2 to the CNS has been explored by several authors, who have proposed different mechanisms (Brann et al., 2020; Chen R. et al., 2020; Natoli et al., 2020; Ueha et al., 2020; Wu Y. et al., 2020). Hence, it is crucial to understand the role of the classical sensory pathways that are linked to the cranial nerve functions in COVID-19. Nonetheless, it is also important to determine any alternative routes that SARS-CoV-2 may take to reach the CNS, including those related to the ENS.

SARS-CoV-2 RNA and its viral particle have been found in the feces of patients with COVID-19 (Chen Y. et al., 2020), with sustained viral RNA shedding even after the end of symptoms. These findings suggest the existence of a fecal-oral transmission route (Wölfel et al., 2020). The SARS-CoV and MERS-CoV viruses remain viable in sewage water for a few days and can spread through fecal-oral transmission (Wang et al., 2005; Chan et al., 2011; Van Doremalen et al., 2013). It has been suggested that SARS-CoV-2 is also transmitted through this route (Yeo et al., 2020). In addition, it is possible that binding to ACE2 receptors is also a critical determinant of infectivity that must be considered when analyzing the pathophysiology of COVID-19 (Wrapp et al., 2020; Yeo et al., 2020). The small intestine and colon epithelial cells, in particular the enterocytes, strongly express the ACE2 receptor, displaying even higher expression levels than the lung (Lamers et al., 2020; Liang et al., 2020; Zhou J. et al., 2020). In addition, the same study indicated that the colon neural ganglia also express this receptor. Moreover, viral mRNA has been found in the feces of patients with COVID-19 (Chen Y. et al., 2020; Xiao et al., 2020), suggesting that SARS-CoV-2 may cause not only respiratory but also enteric infection. Therefore, it is equally important to explore the contribution of the ENS to COVID-19. Possible involvement of the ENS and the so-called gut-brain axis is further discussed below.

## Possible Mechanisms Associated With SARS-CoV-2 Neuroinvasion

Some evidence suggests that the respiratory effects of COVID-19 might be at least partially associated with the neuroinvasive potential of SARS-CoV-2 (Li Y. C. et al., 2020). As mentioned above, neurological symptoms have been reported in patients with COVID-19 (Wang D. et al., 2020). More importantly, there is strong evidence that rather than being restricted to the respiratory system, coronaviruses also invade the CNS, which could be associated with significant brain damage, different types of neurological diseases, and damage of the medullary cardiorespiratory center which could in turn also affect the respiratory function (Gautier and Ravussin, 2020; Giacomelli et al., 2020; Mao et al., 2020; Poyiadji et al., 2020; Wang D. et al., 2020). Some past studies also indicated the presence of SARS-CoV particles located in brain neurons in samples from infected individuals (Ding et al., 2004; Gu et al., 2005; Li et al., 2016).

Previous experimental models may help to understand the neuromechanisms related to COVID-19. However, caution is essential in evaluating the results of studies that demonstrated the dissemination of SARS-CoV and MERS-CoV to the CNS. Those studies used ACE2 (in the case of SARS-CoV) or DPP4 (in the case of MERS-CoV) transgenic mice, without demonstrating viral spread to the brain of wild-type mice. Therefore, these transgenic models resolve issues related to the infection in wild-type mice by SARS-CoV or MERS-CoV, such as the differences in the structure of mouse and human ACE2 proteins that reduce the tropism of SARS-CoV to mouse tissues (Natoli et al., 2020). A transgenic model was generated to resolve this issue (Netland et al., 2008). In that study, a vector that carried a human ACE2-coding sequence was introduced into wild-type mice (hACE2) under control of the human cytokeratin 18 (K18) promoter (McCray et al., 2007). Upon SARS-CoV infection, the K18-hACE2 transgenic mice showed viral spread through the olfactory pathway, which reached the subcortical and cortical regions (Netland et al., 2008). Some studies performed with transgenic mice showed that both SARS-CoV and MERS-CoV when administered intranasally, may gain access to the brain tissue and rapidly spread toward specific brain areas such as the brainstem and thalamus (Li et al., 2016; Chen Y. et al., 2020). This route of infection could involve the olfactory pathway (Giacomelli et al., 2020). Remarkably, the brainstem, an area that receives the primary afferents of the majority of the cranial nerves and that encloses the medullary cardiorespiratory center, seems to be among the brain regions that are most infected by SARS-CoV and MERS-CoV (McCray et al., 2007; Netland et al., 2008; Li et al., 2016; Li Y. C. et al., 2020). This suggests that infection by SARS-CoV-2 also causes significant changes in many CNS areas, which would probably be reached through routes involving primary afferents of cranial nerves. Nonetheless, the specific route or set of routes by which coronaviruses infect the CNS has not been completely clarified. Lymphatic/hematogenous spread does not seem to be a possibility, especially considering that virtually no viral particle has been detected in the non-neuronal cells of the infected brain areas in cases of SARS-CoV or MERS-CoV (Ding et al., 2004; Gu et al., 2005). This observation also supports the hypothesis that the access of coronaviruses to the CNS would involve peripheral (e.g., cranial) nerves, through synapse-connected neural circuits (Andries and Pensaert, 1980; Matsuda et al., 2004; Li et al., 2012, 2013; Li Y. C. et al., 2020).

## Potential Neural Circuits Related to the Migration of SARS-CoV-2 From the Periphery to the CNS

**Figure 1:**
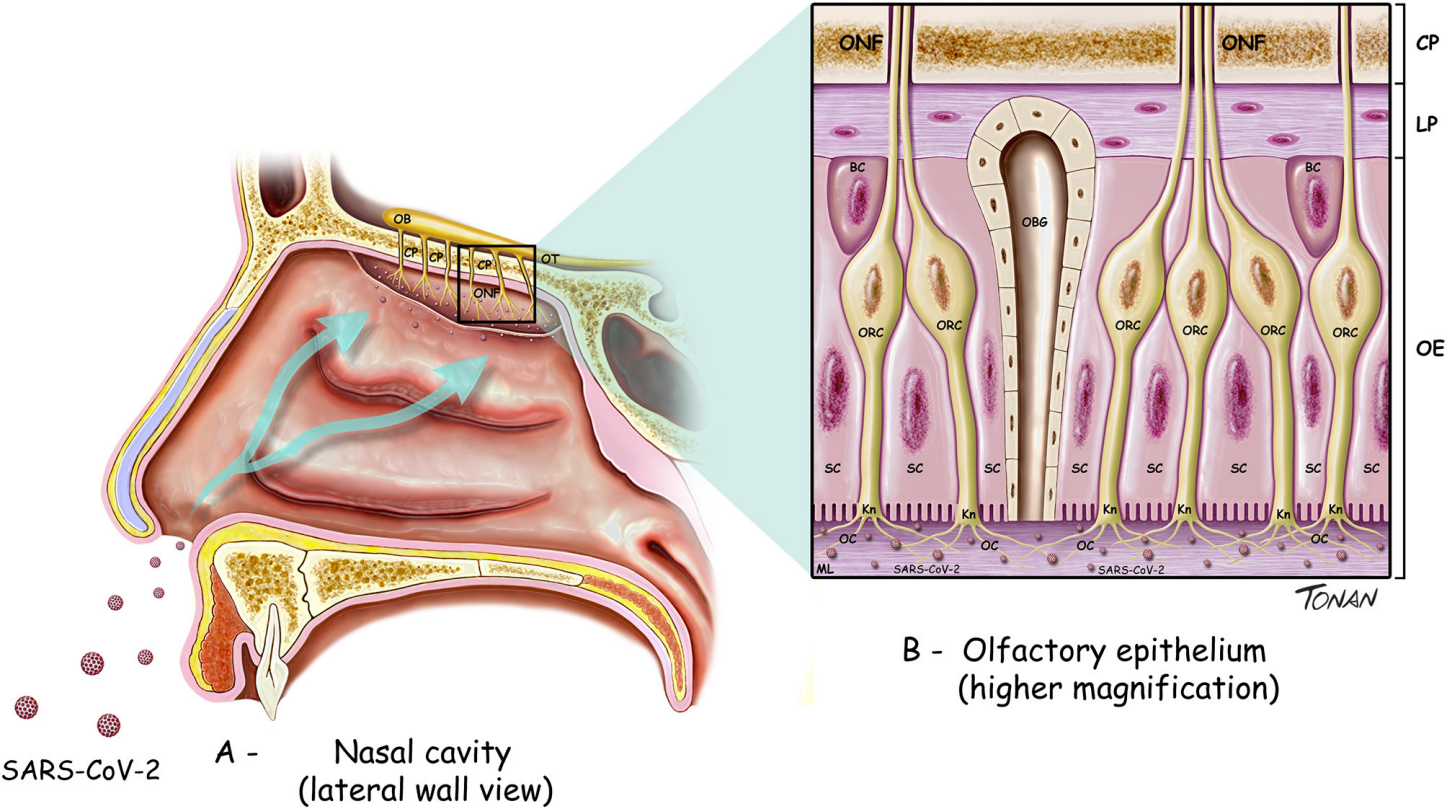
Diagram illustrating the cell types found in the olfactory bulb and part of the olfactory pathway. **(A)** OB, olfactory bulb; OT, olfactory tract; ONF, olfactory nerve fibers; CP, cribiform plate of ethmoid bone. **(B)** ONF, olfactory nerve fibers; CP, cribiform plate of ethmoid bone.; LP, lamina propria; OE, olfactory epithelium; BC, basal cells; SC, supporting (sustentacular) cells; OBG, olfactory Bowman's gland; ORC, olfactory receptor cells; Kn, olfactory Knobs; OC, olfactory cilia.

**Figure 2:**
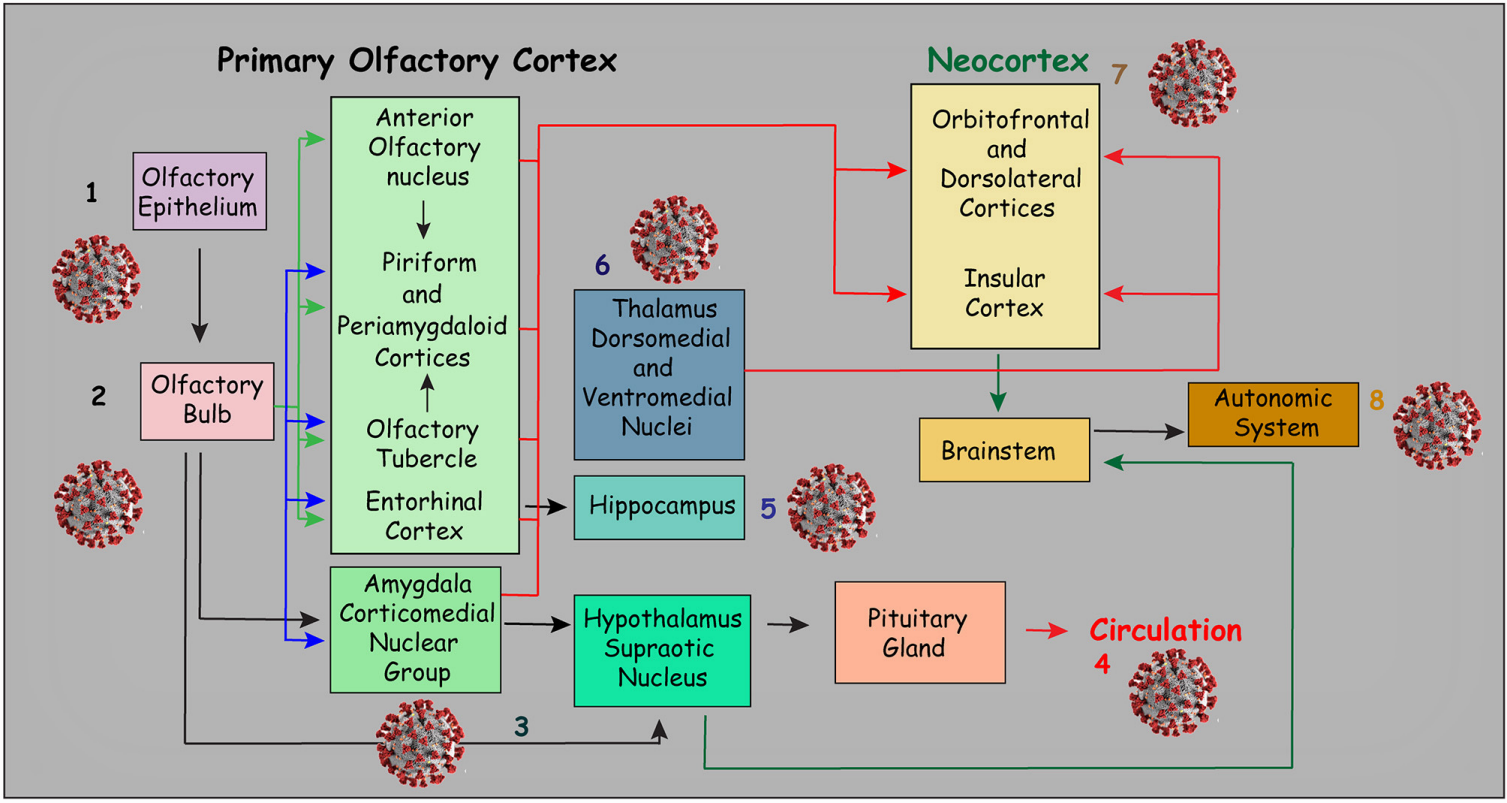
Diagram illustrating the main connections of the primary olfactory cortex. Viral sketches represent the possible symptoms related to the area of impairment: Loss of smell is probably the most evident symptom (1 and 2). However, through direct and indirect connections, the olfactory pathway may be related to several neurologic symptoms. For instance, the connections of the olfactory pathway with the amygdala nuclei may be related to emotional impairment. In addition, other central nervous system changes and neurologic symptoms that may be related to direct or indirect connections of the olfactory pathway are illustrated in this figure. For example, indirect connections to some hypothalamic nuclei, especially the supraoptic nucleus (3) may affect neuroendocrine control, which may, in turn, affect the function of the pituitary gland (4). Moreover, connections to the hippocampus (5) may result in memory impairment. On the other hand, indirect connections to some thalamic nuclei may be associated with sensory and movement impairment (6) while the connections to the frontal and insular cortices (7) may be associated with cognitive impairment. Indirect connections with the brainstem may also result in autonomic nervous system impairment (8). Not surprisingly, the ACE receptor has been found in several of the areas illustrated in this figure (Chen R. et al., 2020). This figure has been adapted from a previous work Devere (2017).

The olfactory pathway is considered one of the strongest candidate routes for SARS-CoV-2 to reach the CNS. Although the evidence is sparse, the current literature points in this direction. The olfactory pathway starts at the olfactory epithelium localized in the upper part of the nasal cavity, i.e., the olfactory mucosa. Olfactory receptors are small bipolar neurons that contain dendrites emerging from one end of their cell bodies, and axons emerging from the opposite end. The unmyelinated and very thin axons of the olfactory receptors collect into a series of small bundles that cross the holes located in the cribriform plate of the ethmoid bone, to end at the olfactory bulb. These small bundles collectively comprise the olfactory nerve. Unlike other mammalian nerves, the olfactory receptors are replaced throughout life, and new receptors arise from undifferentiated basal cells located in the basal epithelium. This may be important, considering the duration of the olfactory symptoms related to COVID-19. The synapse between the first and second neurons of the olfactory pathway occur at the olfactory bulb on each side, specifically at the olfactory glomeruli (Figure 1). Axons of the second-order neurons of this pathway follow a central route into the olfactory tract. The primary central projections of the olfactory bulb are the olfactory nucleus, olfactory tubercle, piriform cortex, amygdala, peri-amygdaloid cortex, insula, and the anterior part of the parahippocampal gyrus (entorhinal cortex) (Nolte, 2009). Of particular interest are the absence of a thalamic relay (although other important connections are established by the olfactory pathway) and the presence of only two neurons in this pathway. However, its cortical targets might be vital in the context of COVID-19. Possible changes in the activity of cortical targets of the olfactory pathway induced by SARS-CoV-2 could, at least in part, explain the neurological symptoms reported by patients with COVID-19 (Figure 2). For instance, the corticomedial amygdala is connected to the anterior preoptic and ventromedial nucleus of the hypothalamus. On the other hand, the central nucleus of the amygdala is connected to the lateral part of the hypothalamus (Standring, 2008). The amygdala has two projections to the hypothalamus: the stria terminalis, a long bundle that follows the caudate nucleus, and a shorter pathway, called ventral amygdalofugal. In addition, it is important to remember the olfactory nerve's connections to the hypothalamus, considering the critical role of the hypothalamus in controlling body temperature and its connections to the autonomic nervous system (Paxinos and Mai, 2004). Thus, an infection by SARS-CoV-2 that affects the neurons and synapses along these routes could compromise these essential functions. In addition, taste depends on integration at the frontal lobe of different stimuli that are not only gustatory. Unsurprisingly, the primary olfactory cortex sends information to the frontal lobe via direct projections, or through a relay in the dorsomedial nucleus of the thalamus (Nolte, 2009). All these connections are important to understand the crosstalk between the olfactory pathway and many cortical and subcortical structures, and therefore their potential importance for SARS-CoV-2. Some studies have analyzed the possible involvement of the olfactory pathway in the spread of SARS-CoV-2 from the periphery to the CNS, as well as the contribution of the cells located in the olfactory mucosa to the olfactory disturbances that are frequently found in patients with COVID-19.

By analyzing data from available brain transcriptome databases, one interesting study evaluated the expression of ACE2 in human and mouse brains (Chen R. et al., 2020). This study also scrutinized the expression of ACE2, along with the main structures of the olfactory system. Strikingly, the distribution of ACE2 was similar in both the mouse and human brain. However, high expression of ACE2 was found in the mouse olfactory bulb, whereas in human brains, ACE2 was more highly expressed in the piriform cortex. Although no data were available regarding the expression of ACE2 in the human olfactory bulb, these findings, at least indirectly, support the concept that the invasion of the CNS by SARS-CoV-2 might occur through the olfactory system. It has also been suggested that the unique characteristics of the olfactory nerve, the olfactory bulb, and the other components of the olfactory pathway may help to explain the importance of this route for the dissemination of SARS-CoV or SARS-CoV-2 to the CNS (Natoli et al., 2020). Some unique characteristics of the olfactory pathway include the presence of olfactory receptor cells in the mucosa, in this case, the olfactory epithelium, the presence of only first and second neurons that synapse at the olfactory glomeruli, the ipsilateral central projections, and the absence of a thalamic relay (Nolte, 2009). In addition, high expression of ACE2 in some hypothalamic nuclei was found in the human brain (Chen R. et al., 2020). As already mentioned, the hypothalamus may be indirectly linked to the olfactory pathway. Nonetheless, the specific mechanisms through which SARS-CoV-2 reaches the CNS through the olfactory pathway are still not clear. Interestingly, the same study showed high expression of ACE2 in some important brain areas such as brain ventricles and substantia nigra but also areas directly or indirectly related to the olfactory pathway, including the amygdala, the hippocampus, some hypothalamic nuclei and the frontal cortex (Chen R. et al., 2020). Some of these regions and connections are illustrated in Figure 2.

One study suggested that SARS-CoV-2 may adhere to different regions of the respiratory system (e.g., nasal cavity, pharynx, nasopharynx, larynx, and trachea). When the virus adheres to the nasal cavity, it can reach the olfactory epithelium, infect the olfactory neurons located in the olfactory epithelium (Figure 1), and then reach the CNS via the olfactory nerve and the related neural circuits (Li Z. et al., 2020). However, other studies have provided more detailed information on SARS-CoV-2 from the olfactory mucosa to the CNS. Through analyses of RNA-seq libraries from the human olfactory neuroepithelium, one study reported the expression of both ACE2 and TMPRSS2 in the olfactory sustentacular cells, microvillar cells, Bowman's glands, and horizontal basal cells (Fodoulian et al., 2020). Another study used *in-situ* hybridization to analyze the expression of *TMPRSS2* mRNA in the olfactory epithelium (Bilinska et al., 2020), which showed that *TMPRSS2* mRNA was present at high levels in sustentacular cells, very low (if at all) in mature olfactory receptor neurons, and, at a lower level, in horizontal basal cells. The same study assessed the expression of ACE2 by immunohistochemistry, which confirmed the expression of ACE2 in sustentacular cells, but not in receptor neurons or basal cells (Bilinska et al., 2020). According to Fodoulian et al. (2020), sustentacular cells, rather than other positive cells for ACE2 and TMPRSS2 in the olfactory sensory epithelium, are the prime candidates for the origin of SARS-CoV-2-induced anosmia. Supporting this hypothesis is the rapid development of anosmia triggered by SARS-CoV-2, the high amounts of TMPRSS2 and ACE2 transcripts in sustentacular cells, and the critical role played by sustentacular cells in maintaining the integrity of the olfactory neuroepithelium. When the sustentacular cells are lost, the entire neuroepithelium disintegrates, leading to anosmia. Some examples include transient anosmia related to the effects of some chemical agents that affect sustentacular cells, such as 3-methylindole (Miller and O'bryan, 2003), the antithyroid drug methimazole (Bergström et al., 2003) or nickel sulfate NiSO(4) (Jia et al., 2010). Another study analyzed bulk and single-cell RNA-Seq datasets of humans and mice to determine the cell types in both the olfactory epithelium and the olfactory bulb that express cell-entry molecules related to SARS-CoV-2 infection. Based on the absence of the ACE2 and TMPRSS2 genes in the olfactory sensory neurons as well as in the olfactory bulb, contrasting with their presence in supporting cells, stem cells, and perivascular cells, the authors concluded that anosmia and other olfactory disturbances found in patients with COVID-19 is associated with non-neuronal cells (Brann et al., 2020). Therefore, an alternative mechanism, not involving the direct infection of olfactory neurons, must be considered when interpreting the presence of anosmia in patients with COVID-19. Another study used immunohistochemistry and gene analyses to determine the presence of ACE2, TMPRSS2, and Furin in the respiratory mucosa, olfactory mucosa, and olfactory bulb of both human and mouse tissues (Ueha et al., 2020), which showed that ACE2 was widely expressed in the respiratory mucosa, olfactory mucosa, and olfactory bulb. ACE2, TMPRSS2, and Furin were co-expressed in the respiratory mucosa (e.g., respiratory epithelium and subepithelial glands) and in the olfactory mucosa, particularly in the supporting cells of the olfactory epithelium and Bowman's glands. However, the olfactory receptor neurons of the olfactory mucosa were positive for ACE2 but almost negative for TMPRSS2 and Furin. Olfactory bulb cells strongly expressed ACE2, weakly expressed Furin, and did not express TMPRSS2. The authors of this study concluded that odor transduction can be impaired by neuronal dysfunction, considering the co-expression of ACE2 and TMPRSS2 in the olfactory nerve bundles. Nevertheless, they suggested that it is unlikely that SARS-CoV-2 directly damages the olfactory receptor neurons, since these cells seem to express ACE2, but not TMPRSS2 or Furin. According to the authors, this absence of TMPRSS2 and Furin expression by olfactory receptor neurons could determine an early recovery of anosmia in patients with COVID-19. However, this concept must be further explored.

Conversely, Fodoulian et al. (2020) demonstrated that human horizontal basal cells express ACE2 and TMPRSS2 at low levels. Horizontal basal cells are progenitors that divide throughout adult life and continually replace sensory neurons (Durante et al., 2020). This suggests that olfactory sensory neurons differentiated from infected horizontal basal cells may be infected by SARS-CoV-2, and via a transsynaptic route, this virus migrates through the olfactory bulb to reach the olfactory cortex.

Importantly, not only the olfactory system but also other routes must be considered when exploring the route of entry into the CNS by SARS-CoV-2. For instance, high expression of ACE2 in both the central glial substance and in the cerebrospinal fluid (CSF) has also been found in the human brain (Chen R. et al., 2020). This is important since it provides additional routes that might be potentially used by SARS-CoV-2 to reach the CNS. In addition, the possible contribution of other sensory pathways to this process must be evaluated in detail (Li Z. et al., 2020).

Flavor perception depends on the integrity of the facial, glossopharyngeal, and vagus nerves. Each of these nerves encompasses pseudo-unipolar neurons. The cell bodies of each of these neurons are located within specific peripheral ganglia, as follows: geniculate ganglion (facial nerve), nodose ganglion (vagus nerve), and petrosal ganglion (glossopharyngeal nerve). However, the synapses between the first- and second-order neurons of all these three nerves occur at a single nucleus, the nucleus of the solitary tract (NST), located in the medulla, and are considered to be the main visceral nucleus of the brainstem. From the NST, nerve fibers travel through the ipsilateral central tegmental tract to the medial part of the ventral posteromedial (VPM) nucleus of the thalamus, where a synapse with third-order neurons takes place (Nolte, 2009). Finally, these neurons project to the gustatory cortex, located in the insula and the medial surface of the frontal operculum (Nolte, 2009), which may also be potential CNS targets of SARS-CoV-2. Regarding the possible invasion of brainstem nuclei by SARS-CoV-2, the importance of the NST and the nucleus ambiguous (NA) must be emphasized. The NST receives sensory input from both chemoreceptors and mechanoreceptors in the respiratory tracts. On the other hand, the efferent fibers from the NA and the NST innervate the airway smooth muscles, blood vessels, and glands (Standring, 2008). These neuroanatomical relationships, together with the proximity of the NST and the NA with the medullary cardiorespiratory center, suggest that the death of patients with COVID-19 might be associated with damage to the medullary cardiorespiratory center. Furthermore, considering a previous finding that the median time from the first symptom to dyspnea is around 5 days, followed by 7 days to hospital admission, and 8 days to intensive care (Wang D. et al., 2020), it has been suggested that the latency period of COVID-19 would be sufficient for the virus to enter and damage the neurons in the cardiorespiratory center, located in the brainstem (Li Y. C. et al., 2020).

On the other hand, the general sensory inputs from the nasal cavity (respiratory mucosa) and oral cavity are carried out by the primary afferents of the trigeminal nerve. Trigeminal neurons are also classified as pseudo-unipolar and have their cell bodies located in the trigeminal (Gasserian) ganglion. In the trigeminal pathway, the synapses between the first- and second-order neurons occur at the trigeminal brainstem sensory nuclear complex (TBSNC) that extends through a large region of the brainstem (Paxinos and Mai, 2004; Standring, 2008; Nolte, 2009). These neurons then project to the VPM nucleus of the thalamus, where synapses occur with neurons that, in turn, project to the primary sensory cortex (S1). All these anatomical descriptions indicate at least four pathways by which SARS-CoV-2 could act at the CNS level. Of particular interest is the proximity between the TBSNC/NST and the cardiorespiratory center, all located in the medulla.

Regarding the ENS, most of its neurons involved in motor function are located within the myenteric plexus, although some primary afferent neurons reside in the submucous plexus. As in all NS involved in sensory-motor control, the ENS includes primary afferents. Enteric neurons receive input from ascending interneurons (which contain opioid peptides) as well as from descending cholinergic neurons (Costa et al., 2000). Considering the several particularities of the ENS (Coelho-Aguiar et al., 2015), the possible role of this system in COVID-19 will be discussed separately.

## Potential Contribution of the ENS to SARS-CoV-2 Mechanisms and the COVID-19 Gastrointestinal Symptoms

Gastrointestinal symptoms have been reported in ~10% of the patients hospitalized and diagnosed with COVID-19, and abdominal pain may also occur (Wang D. et al., 2020). Xiao et al. (2020) found that 53% of 73 hospitalized patients with COVID-19 had viral RNA in their stools. Approximately 23% of these patients still had positive results in their stools, even after their respiratory samples became viral RNA-negative (Xiao et al., 2020). A recent *in-vitro* study of epithelial-cell infection demonstrated that SARS-CoV-2 productively infects human gut enterocytes (Lamers et al., 2020). In this study, human intestinal organoids were readily infected by both SARS-CoV and SARS-CoV-2, as demonstrated by confocal and transmission electron microscopy. After 60 h, SARS-CoV-2 induced the production of a large number of apoptotic cells (Lamers et al., 2020). The same study also showed that mature enterocytes express 300 times more ACE2 (Wan et al., 2020). As previously described, binding to the ACE2 receptor has been considered an important aspect of COVID-19 infectivity. A structural *in silico* analysis that predicts higher effectiveness in the use of SARS-CoV-2 human ACE2 when compared to the 2003 SARS-CoV strain supports this idea (Wan et al., 2020). Furthermore, biophysical and structural evidence demonstrated a higher affinity of SARS-CoV-2 S protein to ACE2 (~10 to ~20 fold increase when compared to SARS-CoV S protein) (Wrapp et al., 2020). However, proliferative enterocyte progenitors appeared to be the primary targets of gut infection (Lamers et al., 2020). This finding suggests that even low levels of ACE2 may be sufficient for viral entry, at least in these types of cells (Lamers et al., 2020).

In addition, as shown by an mRNA sequence analysis, SARS-CoV-2 infection causes the expression of a wide range of cytokines and interferon-stimulated genes (ISGs), which are attributed to type I and III interferon responses. Notably, the induction of these genes by SARS-CoV occurred at a much lower level (Lamers et al., 2020). A further study that also used organoids showed, through immunofluorescence, the expression of ACE2 in human intestinal organoids, and also the presence of the TMPRSS2 protein (Zhou J. et al., 2020). Based on the high sequence homology of horseshoe bats to SARS-CoVs, a bat origin of SARS-CoV-2 has been proposed (Zhou P. et al., 2020). This information supported the study of Zhou J. et al. (2020) to establish a model to study the gut cells of bats. With this model, the authors compared human and bat organoids. Enteroids derived from intestinal cells of *Rhinolophus sinicus* bats showed ACE2 and TMPRSS2 proteins similar to the human homologs. In addition, they were susceptible to productive SARS-CoV-2 infection. In the same study, the infectious virus was isolated from the stool of a patient with COVID-19 who had diarrhea (Zhou J. et al., 2020). Altogether these findings strongly suggest that the human intestinal tract serves as an alternative route for SARS-CoV-2 infection.

To fully understand the enteric system's contribution to the pathophysiology of SARS-CoV-2 infection, the importance of the gut-brain axis should be considered. The so-called gut-brain axis is highly important in many disorders that affect the CNS, and several recent studies have shown the relevance of this communication for the clinical manifestation and even for the genesis of numerous diseases (Coelho-Aguiar et al., 2019). This communication would make it possible to stimulate specific centers in the CNS (Coelho-Aguiar et al., 2015). In addition, Parker et al. (2020) showed that viral transmission via neurons from the ENS to the CNS is possible both by anterograde and retrograde pathways and that both routes usually reach the same brain sites. As mentioned above, a classical study showed that when inoculated in the orofacial region of suckling piglets, HEV first reaches the upper airway, lungs, and small intestine (Andries and Pensaert, 1980). Also, during the incubation period, viral antigens were detected by immunofluorescence in some peripheral ganglia, such as the inferior vagal ganglion and the intestinal nervous plexuses of the ENS. After that, the HEV spread to the brainstem and was later also found in other regions of the CNS. Indeed, this virus accessed the spinal cord through a route that uses retrograde motor neuron transport from the gut. Infection was also detected in the intestinal nervous plexuses, and the transmission was demonstrated to occur through transsynaptic migration of the HEV (Andries and Pensaert, 1980). Based on this, it has been suggested that, upon infection of enterocytes by coronaviruses, ENS cells would also be infected and transmit the virus to the CNS. An alternative possibility is that this virus causes vagal stimulation of the NST, similar to what takes place in infection by other enteric viruses such as the rotavirus. In these cases, after enterocytes are infected, a rotavirus-encoded protein stimulates the secretion of serotonin (5-HT) by the enterochromaffin cells of the gut, which in turn stimulate neurons of the myenteric ganglia and vagal nerves that project to areas of the CNS (Crawford et al., 2017). Therefore, it is tempting to speculate that the vast network that connects the ENS to the CNS (Standring, 2008) may also be involved in SARS-CoV-2 NS infection and may be associated with the gastrointestinal symptoms reported by patients diagnosed with COVID-19 (2003). However, there is little evidence for the specific involvement of ENS cells in COVID-19, and further investigation is needed.

## Neuro-Immune Role in COVID-19

The significant lung damage seen in patients with COVID-19 has been, in part, associated with a cytokine dysregulation known as “cytokine release syndrome” or “cytokine storm” (Chen G. et al., 2020; Zhang et al., 2020). This so-called “cytokine storm” takes place when the innate and adaptive immune systems, release aberrant levels of cytokine and other factors in an attempt to control the infection, leading to tissue injury or even acute organ failure (Yang et al., 2010; Brune et al., 2015). Several studies have correlated this inflammatory response to patients critically ill with COVID-19 (Leyva-Grado et al., 2010; Chen N. et al., 2020; Huang et al., 2020; Ruan et al., 2020; Wu C. et al., 2020; Zhou F. et al., 2020). The prevalence of the pro- and anti-inflammatory cytokines interleukin receptor-2 (IL-2R), interleukin-6 (IL-6), tumor necrosis factor α (TNF-α) and IL-10 in severe and moderate cases of COVID-19 has also been studied (Chen G. et al., 2020). Notably, both SARS-CoV and SARS-CoV-2 can encode viral proteins with immunomodulatory activity, modifying the host's interferon response and enabling the activation of a multiprotein complex termed inflammasome (Fung et al., 2020).

The neuro-immune pathway functions bidirectionally, where afferent neurons respond to immune signals at the periphery, and the efferent neurons promote the interaction between the brain and the periphery. This inflammatory reflex has been discussed (Leyva-Grado et al., 2010; Huang et al., 2020), and the neurological aspects of SARS-CoV-2 are emerging. Increased data pointing to the involvement of the CNS in COVID-19 related symptoms led our group to inquire about how the neuro-immune response could modulate host immunity and lung inflammation. Remarkably Leyva-Grado et al. (2010), using an intranasal mouse model of influenza, observed that viral invasion reaching the CNS through the olfactory bulb modulates brain cytokines in the brainstem, which could be related to elevation in the body temperature as well as disease progression. Moreover, it has been previously demonstrated that the transection of the olfactory bulb causes a decrease of TNF-α and IL-1β. The evidence of an exacerbated inflammatory response in severe cases of COVID-19 suggests that patients with olfactory and gustatory dysfunctions have less severe lung function impairments.

One study demonstrated that 45% of patients presented neurological symptoms and had lower lymphocyte counts (Mao et al., 2020). The author also noted that the severely affected subgroup of patients, who presented neurological symptoms, also showed a decrease in lymphocyte count (Mao et al., 2020). This lymphocytopenia seems to be transient (excluding long-term immunosuppression risks) and is probably due to virus-directed destruction of infected cells since the virion was detectable in these cells (Wang X. et al., 2020). Interestingly, lymphocytopenia is being adopted as a severity marker for COVID-19, as described for different groups or subgroups of patients (Leyva-Grado et al., 2010; Chen N. et al., 2020; Liu et al., 2020; Wang D. et al., 2020). Finally, COVID-19 has been associated with a case of the auto-immune disease, Guillain-Barré syndrome, emphasizing the importance of understanding viral-elicited immune responses and their long-term consequences (Virani et al., 2020).

## Important Considerations and Additional Explanations for the Neurological Symptoms Related to COVID-19

An additional explanation for the neurological signs/symptoms related to COVID-19 may be the occurrence of a systemic inflammatory response. Indirect mechanisms, such as hypercoagulability with thrombosis (Yin et al., 2020), due to an inadequate activation pathway of cytokines and the platelet response (Fox et al., 2020), may also occur. Moreover, the existence of axonal peripheral neuropathy and myopathy, probably caused by diffuse vasculitis, was previously identified with SARS-CoV (Tsai et al., 2004). Even though symptoms related to changes in the peripheral nervous system have recently been reported in patients with COVID-19 (Mao et al., 2020), the neuronal impairment related to COVID-19 has not been confirmed. However, it is important to consider the natural course of this disease, which could lead to a mismatch between the clinical, biochemical, and imaging features found during the different stages of COVID-19. For instance, one article reported a case of a young patient diagnosed with COVID-19 who presented meningitis/encephalitis, seizure, and consciousness disturbance, with abnormal findings in brain magnetic resonance imaging (MRI) (Moriguchi et al., 2020). In this case report, COVID-19 was confirmed through polymerase chain reaction (PCR), which was positive for SARS-CoV-2 in the cerebrospinal fluid (CSF), but not in the nasopharyngeal swab (Moriguchi et al., 2020). These findings show that, despite the clinical presentations described, there is still a need for in-depth exploration and identification of the real neurological involvement in COVID-19. In another case report, acute hemorrhagic necrotizing encephalopathy was found in a patient with COVID-19 (Poyiadji et al., 2020). However, a detailed study and proper explanation for several neurological symptoms found in patients with COVID-19 should be conducted in the future.

## Strategies to Study the Neuroinvasive Potential of SARS-CoV-2

Some methods might be proposed to study the effects of SARS-CoV-2 on the CNS. Among the challenges is the development of an appropriate animal model of COVID-19. There are structural differences in mouse ACE2 compared to human ACE2 proteins, and the SARS coronaviruses exhibit poor tropism for mouse tissues. Therefore, transgenic models are important tools for studying the course of COVID-19. Evaluation of the pathogenicity of SARS-CoV-2 in hACE2 (human angiotensin-converting enzyme-2) transgenic mice demonstrated only mild disease in these chimeric animals, and an absence of viral growth in wild-type mice, emphasizing the importance of hACE2 for SARS-CoV-2 infection and replication in this animal model (Amanat and Krammer, 2020; Bao et al., 2020). However, as previously found with MERS and SARS-COV, as discussed above, the pattern of the disease found in transgenic mice is different from the pattern in wild-type mice. The development of a genetically tractable mammalian model to mimic human pathogenic phenotypes alongside a genetic control system of both host and hCoV genomes could facilitate the understanding of the relationship between virus/host genetics and the pathogenic outcomes (Cockrell et al., 2018).

Considering microRNAs (miRNAs) as possible therapeutic agents against SARS-CoV-2, a computational study aiming to identify possible interactions between host-cellular miRNAs and viral genes was undertaken (Saçar Demirci and Adan, 2020). This study demonstrated that viral genes involved in mechanisms such as biogenesis, entrance, replication, and infection are possible targets for host-cellular miRNAs (Saçar Demirci and Adan, 2020). However, two aspects of this miRNA-based therapeutic strategy against viruses must be considered: a single nucleotide mutation within the virus target region could disrupt the interaction with the host-cellular miRNAs, resulting in viral evasion; and caution is advised when manipulating the level of host-cell miRNAs, due to the important physiological functions of these molecules. Modulation of miRNA levels could lead to unexpected side effects (Mallick et al., 2009).

Another important question is the degree to which the virus causes damage to neurons in specific brain regions (e.g., medullary cardiorespiratory center). For this purpose, primary cell cultures would be the most feasible approach. Immunohistochemistry will also be important to compare the findings in humans and animals, using a post-mortem analysis. However, this can only be considered when using specific antibodies for SARS-CoV-2. Another more modern approach is the use of organoids differentiated from adult stem cells (ASCs) to study COVID-19. Organoids are three-dimensional structures grown from ASCs, which recapitulate several features of the organ from which the ASCs that give rise to them derive. A recent study showed that enterocytes could be infected by SARS-CoV and SARS-CoV-2 in human small intestinal organoids (hSIOs), demonstrating the feasibility of this method to study the pathophysiological mechanisms related to COVID-19 (Lamers et al., 2020).

Regarding *in-vivo* human studies, diffusion tensor imaging (DTI or DTI-MRI) is a promising and extremely safe neuroimaging tool that could be used to study the pathophysiology of COVID-19. This technique can be easily used to examine the brains of patients with COVID-19, as well as the brains of experimental models infected with human SARS-CoV-2 that develop characteristic human-equivalent symptoms. DT-MRI is considered the only non-invasive method that permits a full analysis of the microstructural organization of CNS tissues *in vivo* (Jones and Leemans, 2011). Overall, with DT-MRI, it is possible to estimate the magnitude and direction of water molecules in the microstructure of the main brain tracts. This allows researchers to evaluate changes in these structures under different conditions. A detailed study of the modifications that occur in the brainstem tracts has been successfully achieved. Hence, DT-MRI would be a robust tool to use to study the changes that occur in the brainstem and cortical tracts due to COVID-19. DTI studies would allow researchers to study the specific tracts that are potentially damaged in patients with COVID-19. For instance, with this method, it is possible to study the majority of the olfactory pathway as well as the entire trigeminal pathway, including not only the trigeminal nerve and its peripheral branches (e.g., ophthalmic, maxillary, and mandibular branches) and the trigeminothalamic tract and its nuclei, including the nucleus caudalis, but also the main sensory nucleus and the mesencephalic nucleus. DTI will also allow researchers to study the NST and the NA, as well as their relationship to the medullary cardiorespiratory center. In addition, changes that may occur in different neural pathways related to COVID-19, especially when comparing the specific tracts of COVID-19 patients with the same tracts of healthy individuals, can be evaluated. All tracts associated with COVID-19 related neuroinvasion and discussed in this review can be evaluated with DTI (Wiegell et al., 2003; Granziera et al., 2006; Lutz et al., 2008; Wilcox et al., 2013), including the olfactory system (Milardi et al., 2017).

Neuroimaging techniques such as DT-MRI seem to be particularly compatible with artificial intelligence (AI) data mining, which could improve the diagnosis and prediction of the risk factors involved in the development of more severe forms of COVID-19. To accelerate research in this area, thus providing more robust scientific evidence to combat COVID-19, the White House, in collaboration with research institutes and technology companies, issued a global call for research on COVID-19 on March 16, 2020 (Alimadadi et al., 2020). AI might be used for research in different areas to develop more rapid diagnostic methods with reasonable accuracy and reduce health professionals' exposure to the virus, reducing contamination risks. This approach could be decisive in expediting and reducing the cost of screening for effective treatments to combat COVID-19, and to increase understanding of its pathogenesis. The combination of classical experimentation with other technologies is crucial to determine the neuroinvasion potential and related implications of SARS-CoV-2.

## Conclusions

The effects of COVID-19 on the human NS have been inadequately explored. It is essential and urgent to elucidate the degree of NS involvement in COVID-19, as well as determine the neural circuits, if any, that are potentially affected by SARS-CoV-2. The participation of the PNS, especially the cranial nerves and ENS, as well as the CNS and related neural networks impacted by COVID-19, should be confirmed. Moreover, the directionality and time course of the viral spread in the body should be established. The integration of different technologies and fields of research should be encouraged.

## Author Contributions

MD, DC, and VM-N conceptualized this manuscript. MD, SD, VA, DC, NR, JC-A, TS, JS, CP, ID'A, PN, and VM-N drafted the manuscript. All authors have reviewed and agree with the publication of this manuscript.

## Conflict of Interest

The authors declare that the research was conducted in the absence of any commercial or financial relationships that could be construed as a potential conflict of interest.
